# A Machine Learning Approach to Personalize Computerized Cognitive Training Interventions

**DOI:** 10.3389/frai.2022.788605

**Published:** 2022-03-08

**Authors:** Melina Vladisauskas, Laouen M. L. Belloli, Diego Fernández Slezak, Andrea P. Goldin

**Affiliations:** ^1^Laboratorio de Neurociencia, Universidad Torcuato di Tella, Buenos Aires, Argentina; ^2^Consejo Nacional de Investigaciones Científicas y Técnicas (CONICET), Ministry of Science, Technology and Innovation, Buenos Aires, Argentina; ^3^Laboratorio de Inteligencia Artificial Aplicada, Instituto de Ciencias de la Computación, Universidad de Buenos Aires, Buenos Aires, Argentina

**Keywords:** computerized games, educational games, individual differences, personalized training, machine learning, educational neuroscience, children

## Abstract

Executive functions are a class of cognitive processes critical for purposeful goal-directed behavior. Cognitive training is the adequate stimulation of executive functions and has been extensively studied and applied for more than 20 years. However, there is still a lack of solid consensus in the scientific community about its potential to elicit consistent improvements in untrained domains. Individual differences are considered one of the most important factors of inconsistent reports on cognitive training benefits, as differences in cognitive functioning are both genetic and context-dependent, and might be affected by age and socioeconomic status. We here present a proof of concept based on the hypothesis that baseline individual differences among subjects would provide valuable information to predict the individual effectiveness of a cognitive training intervention. With a dataset from an investigation in which 73 6-year-olds trained their executive functions using an online software with a fixed protocol, freely available at www.matemarote.org.ar, we trained a support vector classifier that successfully predicted (average accuracy = 0.67, AUC = 0.707) whether a child would improve, or not, after the cognitive stimulation, using baseline individual differences as features. We also performed a permutation feature importance analysis that suggested that all features contribute equally to the model's performance. In the long term, this results might allow us to design better training strategies for those players who are less likely to benefit from the current training protocols in order to maximize the stimulation for each child.

## Introduction

If you encounter an add claiming “*Do you want to improve your memory? With these brain exercises you will see changes in less than x time! Scientifically tested method!*”, what would you think? Unfortunately, to date, there is still not a well described and thoroughly tested method that consistently improves cognitive processes (Dorbath et al., 2011; Au et al., 2014; Buttelmann and Karbach, 2017). Even though over the last 25 years many cognitive or brain training protocols have been put to the test and shown positive outcomes (Goldin et al., 2014; Hsu et al., 2014; Diamond and Ling, 2016; Klingberg, 2016; Buttelmann and Karbach, 2017), many other show the opposite results (Melby-lervåg et al., 2016; Simons et al., 2016; Sala et al., 2019). Hence, a consensus on a “brain training recipe” seems improbable, especially considering the plethora of divergent results (Schwaighofer et al., 2015; Aksayli et al., 2019; Vladisauskas and Goldin, 2021).

Many of the successful examples of how cognitive training can benefit cognition show that stimulation can have a positive impact on Executive Functions (EF) (Anguera et al., 2014; Goldin et al., 2014; Karbach and Unger, 2014; Segretin et al., 2014; Klingberg, 2016; Spencer-Smith and Klingberg, 2017; Wiemers et al., 2019). EF are a group of cognitive processes critical for purposeful, goal-directed behavior, such as the ability to set a goal, to make a plan and stick to it, and to have the flexibility of changing that plan, or even the original goal, if priorities change.

EF mature with the great variety of stimulus and experiences that we undergo from birth and continue to develop throughout life (Colé et al., 2014; Delalande et al., 2020; Johann and Karbach, 2020). While this implies that some aspects of life might act as negative modulators of EF development, such as early vulnerability and prenatal malnutrition (McDermott et al., 2012; McGaughy et al., 2014; Deater-Deckard et al., 2019; Howard et al., 2020), it also signifies that proper life experiences can have positive effects in EF and, consequently, improve academic performance and other general life outcomes. In fact, several studies show that EF development predicts not only school performance, but also a broad array of life outcomes such as mental and physical health (McDermott et al., 2012; Miyake and Friedman, 2013; Diamond, 2020). One of the most frequent cognitive training strategies is to specifically and progressively challenge EF through games, and it has proven to be a powerful positive modulator particularly relevant during childhood, when behavioral and neural plasticity are intense (Sigman et al., 2014; Steinbeis and McCrory, 2020).

Mate Marote is a free-open access cognitive-training software aimed at children between 4 and 8 years old. It consists of a set of computerized games specifically tailored to train and evaluate EF. During the last 13 years several supervised interventions were performed with this software inside the schools. The training has shown to improve EF (e.g., Goldin et al., 2013; Nin et al., 2019) and to elicit transfer to real-world measures of school performance (Goldin et al., 2014).

The training process involves games developed to target individual EF. In each intervention, conducted always in educational settings, a particular set of cognitive skills is trained for 10-to-15 min, one-to-three times a week over several weeks. Performance on these and similar domains is measured before and after the training to test for cognitive changes and to evaluate the effectiveness of the training process. This evaluation of cognition is also assessed with games, which are adaptations of standardized cognitive tests that have been used extensively in the literature, such as a version of the Stroop test to assess inhibitory control and cognitive flexibility (Davidson et al., 2006), or the Child-ANT task to measure attentional networks (Rueda et al., 2004).

Our aspiration is that every child can get the most out of their cognitive training time, and, even though many training games have proven to be effective, the question of who benefits the most and why remains uncertain (Karbach et al., 2017; Albert et al., 2020; Steinbeis and McCrory, 2020). Recent research suggests that the conflicting reports on cognitive training could be caused by individual differences among the subjects that take part in each cognitive training intervention. For instance, the developmental age or the state of cognition prior to the stimulation could be key to understanding why cognitive training does not always work for everyone (Guye et al., 2017). It is therefore an intuitive idea to think about those potential modulators when building training protocols (Green et al., 2019). In this line of research we wonder: how can those differences be taken into account to elicit a better stimulation for every brain? (Jaeggi et al., 2013; Karbach et al., 2017; Shani et al., 2019; Rennie et al., 2020).

In this study, we propose an initial approach towards cognitive training personalization using machine learning algorithms to try to identify subjects that will (or will not) benefit from a certain protocol of cognitive stimulation. In other words, can baseline individual state of cognition predict how much a participant will benefit from a certain intervention?

## Methods

We aimed to take a first step in personalizing interventions by predicting the potential benefits of a cognitive training protocol taking into account baseline individual qualities of the participants (Shani et al., 2019, 2021; Rennie et al., 2020).

## Data

**Figure 1:**
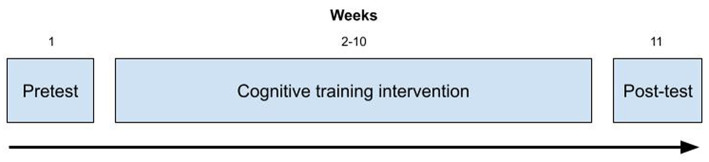
Timeline of the whole experimental procedure. In each session of the intervention children played only one of Mate Marote's games, and performed at least three sessions per week. Standardized evaluations were assessed before (Pretest) and after (Posttest) the cognitive training intervention.

We trained and tested classifiers to predict whether a child would benefit, or not, from a fixed cognitive stimulation strategy. To build these algorithms we used a small dataset from a past intervention performed with Mate Marote's online platform (Goldin et al., 2014). This dataset includes the performance of 73 typically developing 6-to-7 y.o. children (33 girls) in one cognitive training intervention (the same for all children). The intervention involved 3 sequential stages (Figure 1): (A) a Pretest (or baseline) where children's EF and attentional capabilities were measured with a battery of standardized tests, (B) a Training stage where children played several games designed to challenge their EF (referred to as “the cognitive training protocol”); and, finally, (C) a Posttest stage where children's cognition was evaluated again.

During the Training stage, participants played three adaptive computer games aimed at training EF (specifically, working memory, planning, and inhibitory control skills). Children played at their own schools only one game in each 15-min session, and a total of no more than three sessions per week. The three games alternated for all children throughout the intervention. More details of the intervention, together with precise descriptions of the training and evaluation games, are available in Goldin et al. (2014) and Nin et al. (2019).

A week before the beginning of the training and 1 week after the last playing session all children took a battery of standard tests (Pre- and Posttest, respectively). The included tests evaluated: attentional networks (Child-ANT task; Rueda et al., 2004), inhibitory control and cognitive flexibility (The heart-flower task; Davidson et al., 2006), planning (Tower of London task; Phillips et al., 2001) and spatial working memory (Corsi Block Tapping Task; Kessels et al., 2000; Fischer, 2001).

## Model

### Features

Performance in each task depends on the prior state of cognition. As was mentioned earlier, baseline individual differences might include information on how the following cognitive training would work. Which turns each Pretest measure into a possible successful feature. We obtained a total of 12 pretest measures from every participant, and used those values as features to train multiple classifiers (Supplementary Table S1 for a detailed description of every individual feature, which includes attentional resources, inhibition, cognitive flexibility, and planning). The selected features represent different dimensions of each participant's baseline cognition (i.e., obtained during the Pretest). More than one value was obtained from every evaluation in order to perform the expected prediction.

Prior to training the classifier, every individual feature was re-scaled using Sklearn's Robust Scaler (Interquartile range, statistics robust to outliers) and normalized into the range 0–1.

Pairwise comparisons between feature average values at Pretest where made using Mann-Whitney U non-parametric test (McKnight and Najab, 2009).

### Classes

We constructed two classes: “Improved” and “Not improved,” aiming to show whether participants improved, or not, after cognitive training. To consider the existence of improvement after cognitive training, for each feature we calculated a Reliable Change Index (RCI) as was proposed by Jacobson and Truax (1991). The threshold for reliable change is calculated as 1.96 times the standard error of the difference between scores of a measure administered before and after de cognitive training (Pre- and Posttest, respectively). Of the many versions available, we used the method proposed in Estrada et al. (2019) specified as:


(1) $$\begin{array}{l} RCI=\frac{D_{i}}{\sqrt{{\left(S_{pre}\sqrt{1-R_{PrePost}}\right)}^{2}+{\left(S_{post}\sqrt{1-R_{PrePost}}\right)}^{2}}} \end{array}$$

where D_i_ is the individual pre-post difference; S_pre_ and S_post_, the standard deviation at pretest and posttest, respectively; and R_PrePost_ is the internal consistency of the measure. The latter was obtained by calculating Cronbach's alpha following the procedure described in Cronbach (1951).

As every participant completed the same standardized cognitive test twice, at Pre- and Posttest, by comparing the performance metrics between both stages we could evaluate if there were changes after the cognitive training. Hence, for every pair of Pre- and Posttest values we calculated RCI and concluded whether there had been an improvement (if the result was higher than 1.96), a deterioration (if the result was lower than −1.96) or if there was no reliable change between the measures (a result between −1.96 and 1.96).

To obtain the final class for every participant, we counted the amount of improvements and compared it to the amount of measures in which a deterioration was observed. If there were more variables with improved performance, the subject was labeled as “Improved.” If the amount of deteriorated variables was equal or superior to the improvements, subject was labeled “Not improved.”

### The Supervised Algorithms

We performed an hyperparameter tuning with Sklearn's GridSearchCV tool to select the optimal hyperparameter values for a set of 6 classifier algorithms. In alphabetical order, the trained algorithms where: Gradient Boosting (Natekin and Knoll, 2013), K Nearest Neighbors (Laaksonen and Oja, 1996), Multi Layer Perceptron (Suykens and Vandewalle, 1999), Perceptron (Raudys, 1998), Random Forest (Breiman, 2001), and Support Vector Classifier (Lau and Wu, 2003). Afterwards, we compared the accuracy among models, which was calculated within the GridSearch using the optimal hyperparameter values.

### Validation

To obtain a robust accuracy in the test set, for all the 6 algorithms we repeated the training-testing process with the optimal hyperparameter values using a Repeated Stratified K Fold Cross Validation (Refaeilzadeh et al., 2016). Compared to a single train-test split, a cross validation strategy allows to obtain more robust results with a small dataset like ours (as the variance of the data is more evident). Because cross validation tests the model's performance on different train-test splits, it does not have a strong dependency on the instances that belong to each split.

In regular Stratified K Fold Cross Validation, the sample is divided in k equal sized subsamples, each containing roughly the same proportion of the two types of class labels. Of the k subsamples (in this case *k* = 10), a single one is retained as the test set and the remaining are used to train the model (called “training-testing process”). The training-testing process is repeated k times, each time with different subsamples, with each of them used only once as the test set.

The Repeated Stratified K Fold Cross Validation adds an additional step to this process. After performing all the k folds with one set of randomized subsamples, the process restarts with a new set until the training-testing process is complete. The process is repeated n times (in our case, *n* = 20). Hence, we obtained a total of 200 scores (10 folds repeated 20 times) and the results were averaged to produce a single accuracy estimation (i.e., macro average score).

The performance of each model was evaluated using this accuracy score (Trappenberg, 2020). We also obtained the values of recall and precision to get a better idea about the model's learning. In our problem, recall is defined as the proportion of positive cases (“Improved” label) that were detected from the total of real positive cases. Precision refers to the proportion of real positives (labels with a real value of “Improved” predicted as such) from the amount of cases predicted positive. A good algorithm for our problem should prove similarly good in both metrics. We also constructed a receiver operating characteristic curve (ROC) to visualize the model's ability to predict the “Not improved” label, and calculated the area under the curve (AUC; Marzban, 2004) score to reflect the model's ability to predict the “Not improved” class.

As a final validation step, we performed a permutation test for every algorithm. This test is designed to evaluate the significance of a cross-validated score (Venkatraman, 2000). It permutes the targets to generate randomized data (i.e., no relation between features and targets) and calculates the *p*-value against the null hypothesis that features and targets are independent. If *p* < 0.05, we can reject the null hypothesis and assume that they are related, and that the model captures that relationship. To evaluate the significance of each model, we compared the cross-validated score of the algorithm for the randomized data to the score with the original data.

Finally, we selected the algorithm with the higher average accuracy, and performed a permutation feature importance. This analysis provides information on every feature's contribution to the model's prediction. The final weight for every feature is calculated averaging the model's accuracy decrease after randomly permuting the feature values within a testing set. When an important feature is permuted, the score should decrease, while the opposite would happen with a feature that is not very important according to this model's prediction. To obtain robust results with our small dataset, the train-test split was performed with a repeated stratified K fold cross validation as described earlier on this section.

### Data Analysis

All the previous steps were performed in Python 3 language using Jupyter Lab interface. Sklearn library was used to build the machine learning algorithm, and eli5 library to perform the permutation feature importance analysis. Seaborn and Matplotlib were used for data visualization and plotting. Scipy was used to perform statistic analysis.

## Results

After assigning a class to every subject, we obtained a balanced dataset with 73 instances (N_improved_ = 29). We trained 6 classifiers and retrained them with the optimal parameters (individual accuracy values, in Supplementary Table S2). The algorithm that best fitted the data was a support vector classifier (SVC; Lau and Wu, 2003).

**Figure 2:**
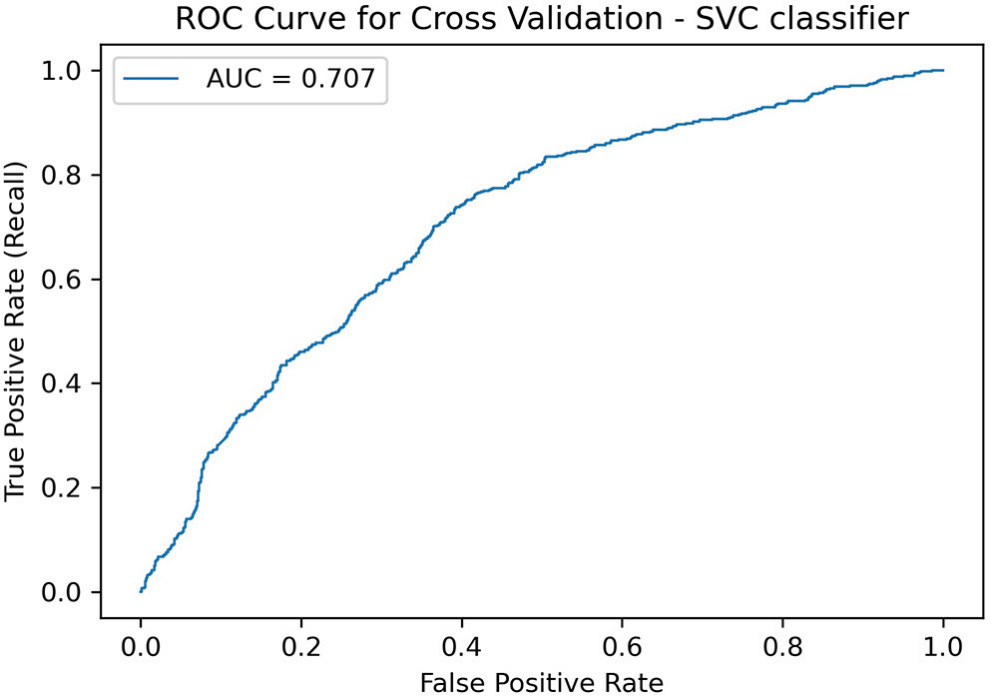
Receiver operating characteristic curve for the SVC model.

The SVC model showed an average accuracy score of 0.67 and AUC of 0.707, which are better than a random baseline performance (final hyperparameter values in Table 1 and permutation importance test result in Supplementary Figure S1). ROC curve in Figure 2. Precision and recall values were similar between classes, as expected from a binary classification on a balanced dataset (Supplementary Table S3). The permutation test result suggests that the SVC final model captures a dependency between the features and the classes (*p* = 0.01 against the null hypothesis that features and targets are independent, see Supplementary Figure S1). The permutation test also suggested that the KNN model was good at the task (accuracy 0.65, *p* < 0.03), although not as good as the SVC model (Supplementary Figure S2).

**Table 1:** Hyperparameter values corresponding to the final SVC model.

**Hyperparameter**	**Value**
C	2.8
class_weight	balanced
kernel	poly

To get a better understanding of the model's predictions, we obtained the permutation feature importance values (Table 2). This exploratory analysis describes which features are the most relevant according to the model's predictions. The standard deviation is large for every feature, suggesting that there are no differences between the feature's contribution in the SVC.

**Table 2:** Permutation feature importance for the final SVC model listed in alphabetical order based on the “Features” column.

**Features**	**Permutation importance**
ANT RT–Alerting Network	0.0349 ± 0.2964
ANT Won %–Alerting Network	0.0274 ± 0.2613
ANT RT–Executive Network	0.0101 ± 0.2613
ANT Won %–Executive Network	0.0150 ± 0.2205
ANT RT–Orienting Network	0.0255 ± 0.2260
ANT Won %–Orienting Network	0.0420 ± 0.2763
Corsi Score	0.0438 ± 0.3038
Stroop RT–Flexibility	0.0370 ± 0.2685
Stroop Won %–Flexibility	0.0040 ± 0.1918
Stroop RT–Inhibition	0.0373 ± 0.2991
Stroop Won %–Inhibition	0.0134 ± 0.2582
TOL Score	0.0477 ± 0.3223

The cognitive training literature presents inconsistent findings on who benefits the most from an intervention, and part of the literature suggests that subjects with the worst Pretest measures are the ones who, at the end, obtain higher cognitive gains (Karbach et al., 2017; Wang et al., 2019). If features differed between classes in the Pretest, they could give us information to approach that open question. The permutation feature importance analysis suggested that all features contribute similarly to the model, but this could be caused by the small size of our dataset. So, we tried to understand the direction of the variance explained by each feature. In other words, we wanted to know if, for each variable, the players who showed a better performance at Pretest were finally classified as “Improved” or as “Not improved.” In order to analyze that, we compared the performance of each of the 12 baseline variables for the “Improved” and the “Not-improved” classes. No difference was significant (Supplementary Table S4).

## Discussion

Our study was meant as a proof of concept in the use of machine learning tools to personalize cognitive training interventions. By focusing specifically on the first two steps suggested by Shani et al. (2019), the final purpose of our research line is to design better, more personalized, training protocols and to contribute to settle the debate on cognitive training efficacy once and for all. Following previous studies which show that some people benefit from a given training protocol more than others (Titz and Karbach, 2014; Karbach et al., 2017), we wanted to know if a training gain could be predicted based solely on previous cognitive traits. Confirmation of this relationship could allow us to prevent some participants from completing a cognitive training protocol that will most likely not improve their EF.

With a small dataset (*N* = 73) from a past intervention performed with a free cognitive training software designed by our group (Goldin et al., 2013, 2014), we aimed to train a set of binary classifiers to predict the outcome of a particular stimulation protocol (i.e., to know whether a participant would benefit or not from it). We were able to train 6 machine learning algorithms, and two of them captured the dependency between features and targets: a *k nearest neighbors classifier* and a *support vector classifier* (SVC). This last algorithm showed the best performance predicting the binary classes (“Improved” or “Not improved”) based on the individual previous cognitive traits. The results of the permutation test indicate that it is able to capture at least a portion of the dependency between the features (individual differences measured in the pretest stage) and the targets (whether a cognitive training was effective). The found accuracy value is moderate (0.67) and can still be improved, but is a very promising result considering the high variability between features (i.e., the individual differences observed in the Pretest).

The AUC value tells how much the model is capable of distinguishing between classes. The AUC result found (0.707) indicates that our model is able to differentiate players that did not show improvements after cognitive training from those who did (“Not improved” and “Improved” classes, respectively).

For this study we built a simple model to understand if we could predict the efficacy of a cognitive training protocol. Although we succeeded, the main limitation of our study is the small dataset. In the future, with more data, we might be able to dissect the “Not improved” class in two subgroups: those who mostly deteriorated (very few participants) and those who remained stable. Furthermore, it would also be interesting to differentiate, within the stable subgroup, those participants who really showed no differences between pre and posttest from those who, on the contrary, improved and deteriorated equally.

To understand the algorithm's prediction mechanism, we obtained the permutation feature importance rank, which showed that no features should be discarded from the SVC model, because there is not enough evidence to say that some of them contribute to the model's accuracy more than the rest. This might be due to the high variability found in the feature's importance, which in turn might be the cause of a small dataset that changes in every iteration, causing the large observed error. Although we cannot discard this explanation, results show that all Pretest measures are valuable in order to predict the efficacy of a cognitive training protocol and, until we can add more data, all cognitive tests prove informative and should be assessed.

Despite, the fact that as already mentioned, we still need to include more data to obtain a more general model for it to be implemented in future interventions, we were able to build a comprehensive baseline model and the results described here have implications for designing personalized cognitive training protocols in order to take into account whether they are going to be effective. For example, the performance of the model predicting the negative class (0.75, Supplementary Table S3) is particularly relevant because it will allow us to identify the subjects that most likely won't improve with one specific protocol. With this information, in the future we could individually target all cognitive training protocols.

Our main priority for the near future is to evaluate if the model does generalize to data from other cognitive training interventions with similar pre/post tests. Thus, we would not only gain insight on the relationship between previous cognitive traits and the training gains, but we may prevent a participant from completing a protocol that will not benefit him/her. Our ultimate goal is to ensure that each child can benefit in the best possible way from the playing time with Mate Marote.

## Data Availability Statement

The raw data supporting the conclusions of this article will be made available by the authors, without undue reservation.

## Ethics Statement

Children's caregivers gave written consent to participate in the study, which was authorized by an institutional Ethical Committee (Centro de Educación Médica e Investigaciones Clínicas, Consejo Nacional de Investigaciones Científicas y Técnicas, protocol no. 486).

## Author Contributions

The planning of this analysis was done by MV together with AG. The data analysis was done by MV, with LB's careful supervision and assistance. The manuscript was written by MV, revised several times by AG and commented thoroughly by LB and DF. All authors contributed to the article and approved the submitted version.

## Funding

This research was supported by Consejo Nacional de Investigaciones Científicas y Técnicas (CONICET). The original data was obtained thanks, also, to the University of Buenos Aires, Human Frontiers, Ministry of Science of Argentina, Centro de Educación Médica e Investigaciones Clínicas, and Fundación Conectar.

## Conflict of Interest

The authors declare that the research was conducted in the absence of any commercial or financial relationships that could be construed as a potential conflictof interest.

## Publisher's Note

All claims expressed in this article are solely those of the authors and do not necessarily represent those of their affiliated organizations, or those of the publisher, the editors and the reviewers. Any product that may be evaluated in this article, or claim that may be made by its manufacturer, is not guaranteed or endorsed by the publisher.

## References

[B1] Aksayli N. Sala G. Gobet F. (2019). The cognitive and academic benefits of cogmed: a meta-analysis. Educ. Res. Rev. 27, 229–243. 10.1016/j.edurev.2019.04.00325010082

[B2] Albert D. W. Hanson J. L. Skinner A. T. Dodge K. A. Steinberg L. Deater-Deckard K.. (2020). Individual differences in executive function partially explain the socioeconomic gradient in middle- school academic achievement. Dev. Sci. 23:e12937. 10.1111/desc.12937 31912610 PMC7392118

[B3] Anguera J. A. Boccanfuso J. Rintoul J. L. Al-Hashimi O. Faraji F. Janowich J.. (2014). Video game training enhances cognitive control in older Adults J.A. Nature 501, 97–101. 10.1038/nature12486 24005416 PMC3983066

[B4] Au J. Sheehan E. Tsai N. Duncan G. J. Buschkuehl M. Jaeggi S. M. (2014). Improving fluid intelligence with training on working memory: a meta-analysis. Psychonomic Bull. Rev. 22, 366–377. 10.1037/e524912015-029 25102926

[B5] Breiman L. (2001). Random forests. Mach. Learn. 45, 5–32. 10.1023/A:1010933404324

[B6] Buttelmann F. Karbach J. (2017). Development and plasticity of cognitive flexibility in early and middle childhood. Front. Psychol. 8, 1040. 10.3389/fpsyg.2017.01040 28676784 PMC5476931

[B7] Colé P. Duncan L. G. Blaye A. (2014). Cognitive flexibility predicts early reading skills. Front. Psychol. 40, 56. 10.3389/fpsyg.2014.00565 24966842 PMC4052802

[B8] Cronbach L. J. (1951). Coefficient alpha and the internal structure of tests. Psychometrika 16, 297–334. 10.1007/BF02310555

[B9] Davidson M. C. Amso D. Anderson L. C. Diamond A. (2006). Development of cognitive control and executive functions from 4 to 13 years: evidence from manipulations of memory, inhibition, and task switching. Neuropsychologia 44, 2037–2078. 10.1016/j.neuropsychologia.2006.02.006 16580701 PMC1513793

[B10] Deater-Deckard K. Mengjiao L. Lee D. King-Casas B. Kim-Spoon J. (2019). Poverty and puberty: a neurocognitive study of inhibitory control in the transition to adolescence. Psychol. Sci. 30, 1573–1583. 10.1177/0956797619863780 31557444 PMC6843747

[B11] Delalande L. Moyon M. Tissier C. Dorriere V. Guillois B. Mevell K.. (2020). Complex and subtle structural changes in prefrontal cortex induced by inhibitory control training from childhood to adolescence. Dev. Sci. 23, 1–12. 10.1111/desc.12898 31469938

[B12] Diamond A. (2020). Executive functions. Handb. Clin. Neurol. 173, 225–240. 10.1016/B978-0-444-64150-2.00020-4 32958176

[B13] Diamond A. Ling D. S. (2016). Conclusions about interventions, programs, and approaches for improving executive functions that appear justified and those that, despite much hype, do not. Dev. Cogn. Neurosci. 18, 34–48. 10.1016/j.dcn.2015.11.005 26749076 PMC5108631

[B14] Dorbath L. Hasselhorn M. Titz C. (2011). Aging and executive functioning: a training study on focus-switching. Front. Psychol. 2, 1–12. 10.3389/fpsyg.2011.00257 22016742 PMC3191350

[B15] Estrada E. Ferrer E. Pardo A. (2019). Statistics for evaluating pre-post change: relation between change in the distribution center and change in the individual scores. Front. Psychol. 9, 2696. 10.3389/fpsyg.2018.02696 30671008 PMC6331475

[B16] Fischer M. H. (2001). Probing spatial working memory with the corsi blocks task. Brain Cogn. 45, 143–154. 10.1006/brcg.2000.1221 11237363

[B17] Goldin A. P. Hermida M. J. Shalom D. E. Costa M. E. Lopez Rosenfeld M. Segretin M. S.. (2014). Far transfer to language and math of a short software-based gaming intervention. Proc. Natl. Acad. Sci. U.S.A. 54, 311–313. 10.1073/pnas.1320217111 24711403 PMC4035955

[B18] Goldin A. P. Segretin M. S. Hermida M. J. Paz L. Lipina S. J. Sigman M. (2013). Training planning and working memory in third graders. Mind Brain Educ. 7, 136–146. 10.1111/mbe.12019

[B19] Green S. C. Bavelier D. Kramer A. F. Vinogradov S. Ansorge U. Ball K. K.. (2019). Improving methodological standards in behavioral interventions for cognitive enhancement. J. Cogn. Enhanc. 3, 2–29. 10.1007/s41465-018-0115-y

[B20] Guye S. De Simoni C. von Bastian C. C. (2017). Do individual differences predict change in cognitive training performance? A latent growth curve modeling approach. J. Cogn. Enhanc. 1, 374–393. 10.1007/s41465-017-0049-9

[B21] Howard S. J. Cook C. J. Everts L. Melhuish E. Scerif G. Norris S.. (2020). Challenging socioeconomic status: a cross-cultural comparison of early executive function. Dev. Sci. 23, 1–9. 10.1111/desc.12854 31077525

[B22] Hsu N. S. Novick J. M. Jaeggi S. M. (2014). The development and malleability of executive control abilities. Front. Behav. Neurosci. 8, 221. 10.3389/fnbeh.2014.00221 25071485 PMC4092375

[B23] Jacobson N. S. Truax P. (1991). Clinical significance: a statistical approach to defining meaningful change in psychotherapy research. J. Consult. Clin. Psychol. 59, 12–19. 10.1037/0022-006X.59.1.12 2002127

[B24] Jaeggi S. M. Buschkuehl M. Shah P. Jonides J. (2013). The role of individual differences in cognitive training and transfer. Mem. Cogn. 42, 464–480. 10.3758/s13421-013-0364-z 24081919

[B25] Johann V. E. Karbach J. (2020). Effects of game-based and standard executive control training on cognitive and academic abilities in elementary school children. Dev. Sci. 23, 1–18. 10.1111/desc.12866 31132209

[B26] Karbach J. Könen T. Spengler M. (2017). Who benefits the most? Individual differences in the transfer of executive control training across the lifespan. J. Cogn. Enhanc. 1, 394–405. 10.1007/s41465-017-0054-z

[B27] Karbach J. Unger K. (2014). Executive control training from middle childhood to adolescence. Front. Psychol. 5, 390. 10.3389/fpsyg.2014.00390 24847294 PMC4019883

[B28] Kessels R. P. C. Zandvoort M. J. E. Postma A. Jaap Kappelle L. de Haan E. H. F. (2000). The corsi block-tapping task: standardization and normative data. Appl. Neuropsychol. 7, 252–258. 10.1207/S15324826AN0704_8 11296689

[B29] Klingberg T. (2016). Neural basis of cognitive training and development. Curr. Opin. Behav. Sci. 10, 97–101. 10.1016/j.cobeha.2016.05.003

[B30] Laaksonen J. Oja E. (1996). Classification with learning k-nearest neighbors. IEEE Int. Conf. Neural Netw. Conf. Proc. 3, 1480–1483. 10.1109/ICNN.1996.549118

[B31] Lau K. W. Wu Q. H. (2003). Online training of support vector classifier. Pattern Recogn. 36, 1913–1920. 10.1016/S0031-3203(03)00038-4

[B32] Marzban C. (2004). The ROC curve and the area under it as performance measures. Weather Forecast. 19, 1106–1114. 10.1175/825.1

[B33] McDermott J. M. Westerlund A. Zeanah C. H. Nelson C. A. Fox N. A. (2012). Early adversity and neural correlates of executive function: implications for academic adjustment. Dev. Cogn. Neurosci. 2(Suppl. 1):S59–S66. 10.1016/j.dcn.2011.09.008 22682911 PMC3408020

[B34] McGaughy J. A. Amaral A. C. Rushmore R. J. Mokler D. J. Morgane P. J. Rosene D. L.. (2014). Prenatal malnutrition leads to deficits in attentional set shifting and decreases metabolic activity in prefrontal subregions that control executive function. Dev. Neurosci. 36, 532–541. 10.1159/000366057 25342495

[B35] McKnight P. E. Najab J. (2009). The Mann-Whitney U test. Corsini Encyclop. Psychol. 531–94. 10.1201/9780429186196-17

[B36] Melby-lervåg M. Redick T. S. Hulme C. (2016). Working memory training does not improve performance on measures of intelligence or other measures of ‘ far transfer ’: evidence from a meta-analytic review. Perspect. Psychol. Sci. 11, 512–534. 10.1177/1745691616635612 27474138 PMC4968033

[B37] Miyake A. Friedman N. P. (2013). The nature and organization of individual differences in executive functions: four general conclusions. Curr. Dir. Psychol. Sci. 21, 1–5. 10.1177/0963721411429458 22773897 PMC3388901

[B38] Natekin A. Knoll A. (2013). Gradient boosting machines, a tutorial. Front. Neurorob. 7, 21. 10.3389/fnbot.2013.00021 24409142 PMC3885826

[B39] Nin V. Goldin A. P. Carboni A. (2019). Mate marote: video games to stimulate the development of cognitive processes. Rev. Iberoamericana de Tecnol. Aprendizaje 14, 22–31. 10.1109/RITA.2019.2909958

[B40] Phillips L. H. Wynn V. E. McPherson S. Gilhooly K. J. (2001). Mental planning and the tower of london task. Q. J. Exp. Psychol. 54, 579–597. 10.1080/713755977 11394063

[B41] Raudys Š. (1998). Evolution and generalization of a single neurone: I. Single-layer perceptron as seven statistical classifiers. Neural Netw. 11, 283–296. 10.1016/S0893-6080(97)00135-4 12662838

[B42] Refaeilzadeh P. Tang L. Liu H. (2016). Cross-validation, in Encyclopedia of Database Systems, eds Liu L. Özsu M. (New York, NY: Springer).

[B43] Rennie J. P. Zhang M. Hawkins E. Bathelt J. Astle D. E. (2020). Mapping differential responses to cognitive training using machine learning. Dev. Sci. 23, 1–15. 10.1111/desc.12868 31125497 PMC7314597

[B44] Rueda R. M. Fan J. McCandliss B. D. Halparin J. D. Gruber D. B. Pappert Lercari L.. (2004). Development of attentional networks in childhood. Neuropsychologia 42, 1029–1040. 10.1016/j.neuropsychologia.2003.12.012 15093142

[B45] Sala G. Aksayli N. D. Tatlidil K. S. Tatsumi T. Gondo Y. Gobet F. (2019). Near and far transfer in cognitive training: a second-order meta-analysis. Collabra Psychol. 5, 1–22. 10.1525/collabra.203

[B46] Schwaighofer M. Fischer F. Bühner M. (2015). Does working memory training transfer? A meta-analysis including training conditions as moderators. Educ. Psychol. 50, 138–166. 10.1080/00461520.2015.1036274

[B47] Segretin M. S. Lipina S. J. Hermida M. J. Sheffield T. D. Nelson J. M. Espy K. A.. (2014). Predictors of cognitive enhancement after training in preschoolers from diverse socioeconomic backgrounds. Front. Psychol. 5, 205. 10.3389/fpsyg.2014.00205 24659975 PMC3952047

[B48] Shani R. Shachaf T. Derakshan N. Cohen N. Enock P. M. McNally R. J.. (2021). Personalized cognitive training: protocol for individual-level meta-analysis implementing machine learning methods. J. Psychiatr. Res. 138, 342–348. 10.1016/j.jpsychires.2021.03.043 33901837

[B49] Shani R. Tal S. Zilcha-Mano S. Okon-Singer H. (2019). Can machine learning approaches lead toward personalized cognitive training? Front. Behav. Neurosci. 13, 64. 10.3389/fnbeh.2019.00064 31019455 PMC6458282

[B50] Sigman M. Peña M. Goldin A. P. Ribeiro S. (2014). Neuroscience and education: prime time to build the bridge. Nat. Neurosci. 17, 497–502. 10.1038/nn.3672 24671066

[B51] Simons D. J. Boot W. R. Charness N. Gathercole S. E. Chabris C. F. Hambrick D. Z.. (2016). Do ‘brain-training’ programs work? Psychol. Sci. Public Interest Suppl. 17, 103–186. 10.1177/1529100616661983 27697851

[B52] Spencer-Smith M. Klingberg T. (2017). Working memory training. Wiley Handbook Cogn. Control 46, 1199–1201. 10.1002/9781118920497.ch28

[B53] Steinbeis N. McCrory E. (2020). Editorial to the special issue on ‘on mechanisms of cognitive training and transfer in development.’ Dev. Sci. 23, 1–4. 10.1111/desc.12932 31837074

[B54] Suykens J. A. K. Vandewalle J. (1999). Training multilayer perceptron classifiers based on a modified support vector method. IEEE Trans. Neural Netw. 10, 907–911. 10.1109/72.774254 18252586

[B55] Titz C. Karbach J. (2014). Working memory and executive functions: effects of training on academic achievement. Psychol. Res. 78, 852–868. 10.1007/s00426-013-0537-1 24389706

[B56] Trappenberg T. (2020). Fundamentals of Machine Learning. Oxford: Oxford University Press.

[B57] Venkatraman E. S. (2000). A Permutation test to compare receiver operating characteristic curves. Int. Biometr. Soc. 56, 1134–1138. 10.1111/j.0006-341X.2000.01134.x 11129471

[B58] Vladisauskas M. Goldin A. P. (2021). The cognitive training quandary : 20 years summarized. COJ Rev. Res. 3, 1–3. 10.1344/joned.v1i1.31628

[B59] Wang C. Jaeggi S. M. Yang L. Zhang T. He X. Buschkhuehl M.. (2019). Narrowing the achievement gap in low-achieving children by targeted executive function training. J. Appl. Dev. Psychol. 63, 87–95. 10.1016/j.appdev.2019.06.002

[B60] Wiemers E. A. Redick T. S. Morrison A. B. (2019). The influence of individual differences in cognitive ability on working memory training gains. J. Cogn. Enhanc. 3, 174–185. 10.1007/s41465-018-0111-2 31595266 PMC6782049

