# Supplementary material for: A Machine Learning Approach to Personalize Computerized Cognitive Training Interventions
Source: Front Artif Intell. 2022 Mar 8;5:788605. doi: 10.3389/frai.2022.788605 (PMC8958026; doi:10.3389/frai.2022.788605)
Supplement: Supplementary file 1 [file Data_Sheet_1.pdf]

Supplementary Material

A machine learning approach to personalize computerized cognitive training interventions

Melina Vladisauskas[1,3*], Laouen M. L. Belloli[2,3], Diego Fernández Slezak[2,3], Andrea P. Goldin[1,3].

[1]Laboratorio de Neurociencia, Universidad Torcuato di Tella, Buenos Aires, Argentina

[2]Laboratorio de Inteligencia Artificial Aplicada, Instituto de Ciencias de la Computación, Universidad de Buenos Aires, Argentina

[3]Consejo Nacional de Investigaciones Científicas y Técnicas (CONICET), Ministry of Science, Technology and Innovation, Argentina

Table 1. Detailed features descriptions listed in alphabetical order based on the "Feature name" column.

Feature name	Feature description	Cognitive test
ANT RT - Alerting Network	For every player we calculated a delta between the average response time on two type of trials: no cue trials – double cue trials	child-ANT
ANT Won % - Alerting Network	For every player we calculated a delta between the amount of correct answers on two type of trials: no cue trials – double cue trials	
ANT RT - Executive Network	For every player we calculated a delta between the average response time on two type of trials: incongruent trials – congruent trials	
ANT Won % - Executive Network	For every player we calculated a delta between the amount of correct answers on two type of trials: incongruent trials – congruent trials	
ANT RT - Orienting Network	For every player we calculated a delta between the average response time on two type of trials: central cue trials – spatial cue trials	
ANT Won % - Orienting Network	For every player we calculated a delta between the amount of correct answers on two type of trials: central cue trials – spatial cue trials	
Corsi Score	For every player we calculated a final score, that was computed by summing the amount of recalled stimulus from every correct trial.	Corsi block-tapping task

Stroop RT - Flexibility	For every player we calculated a delta between the average response time on the third stage of the task: incongruent trials – congruent trials	Heart-Flower Stroop task
Stroop Won % - Flexibility	For every player we calculated a delta between the amount of correct answers on the third stage of the task: incongruent trials – congruent trials	
Stroop RT - Inhibition	For every player we calculated a delta between the average response time on the first two stages of the task: incongruent stage – congruent stage	
Stroop Won % - Inhibition	For every player we calculated a delta between the amount of correct answers on the first two stages of the task: incongruent stage – congruent stage	
TOL Score	For every player we calculated a final score, that was computed by summing the amount of movements required to solve the trial from every correct trial.	Tower of London

Table 2. Tested model's cross validated accuracy score listed in alphabetical order based on the "Model" column.

**Model**	**Accuracy**	**Standard deviation**
Support Vector Classifier	*.67*	*.17*
K Nearest Neighbors	.65	.17
Random Forest Classifier	.59	.06
Gradient Boosting	.57	.18
Multilayer Perceptron Classifier	.57	.18
Perceptron	.52	.14

Table 3. Summarized performance of the SVC model evaluated for both classes based on precision and recall. $N_{improved}$=29. From the 44 not improved subjects, most of the baseline measures remained stable in Postest.

Class	Precision	Recall
Not improved	.72	.75
Improved	.60	.57

Table 4. Pairwise comparisons between training gain groups ("Improved" and "Not improved" after cognitive training) in pretest stage for the 12 assessed variables (listed in alphabetical order based on the "Pretest measure" column).

Pretest measure	Statistic	p-value
ANT RT - Alerting Network	561.0	.194
ANT Won % - Alerting Network	508.0	.069
ANT RT - Executive Network	530.0	.112
ANT Won % - Executive Network	595.5	.316
ANT RT - Orienting Network	542.0	.14
ANT Won % - Orienting Network	560.5	.188
Corsi Score	507.5	.071
Stroop RT - Flexibility	631.0	.471
Stroop Won % - Flexibility	532.0	.115
Stroop RT - Inhibition	573.0	.233

Stroop Won % - Inhibition	598.0	.325
TOL Score	549.5	.16

Figure 1. Permutation test result for the SVC model. The histogram shows the SVC accuracy distribution on the randomized data (200 scores calculated with repeated stratified k fold cross validation). The red line highlights the model's cross-validated score on the original data.

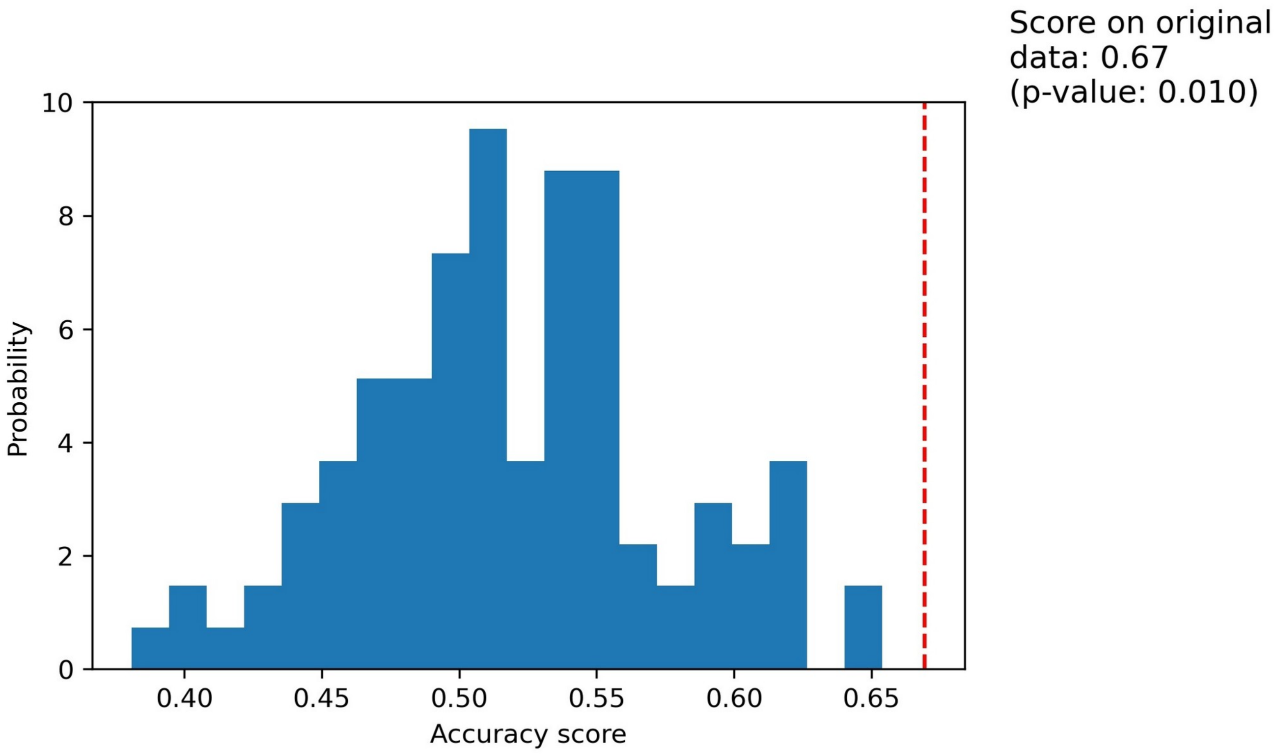

Figure 2. Permutation test result for the KNN model. The histogram shows the KNN accuracy distribution on the randomized data, and the red line highlights the model's cross-validated score on the original data.

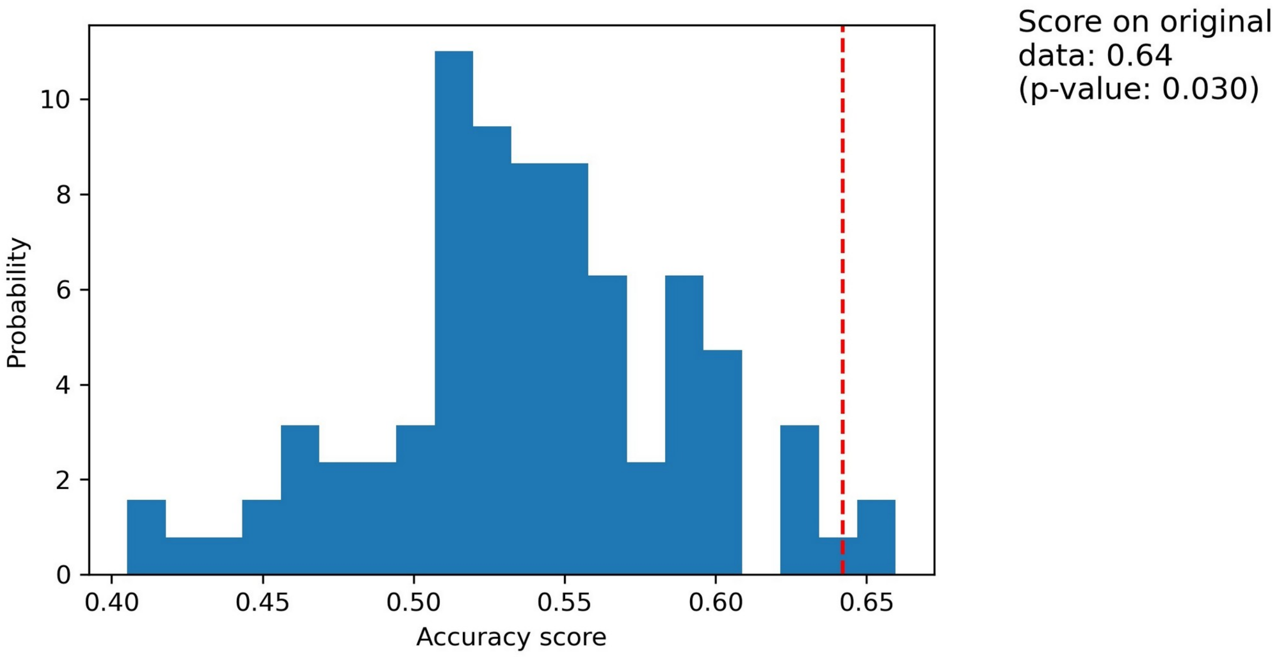

References

Berch, D. B., Krikorian, R., and Huha, E. M. (1998). The Corsi block-tapping task: Methodological and theoretical considerations. *Brain and Cognition, 38(3), 317–338.* https://doi.org/10.1006/brcg.1998.1039

Davidson, M. C., Amso, D., Anderson, L. C., and Diamond, A. (2006). Development of cognitive control and executive functions from 4 to 13 years: Evidence from manipulations of memory, inhibition, and task switching. *Neuropsychologia, 44(11), 2037–2078.* https://doi.org/10.1016/j.neuropsychologia.2006.02.006

Phillips, L. H., Wynn, V. E., McPherson, S., and Gilhooly, K. J. (2001). Mental planning and the Tower of London task. *Q. J. Exp. Psychol. 54, 579–597.* https://doi.org/10.1080/713755977

Rueda, M. R., Fan, J., McCandliss, B. D., Halparin, J. D., Gruber, D. B., Lercari, L. P., and Posner, M. I. (2004). Development of attentional networks in childhood. *Neuropsychologia, 42(8), 1029– 1040.* https://doi.org/10.1016/j.neuropsychologia.2003.12.012
